# A Gastrocolic Fistula Presenting as Chronic Diarrhea

**DOI:** 10.14309/crj.0000000000000326

**Published:** 2020-02-19

**Authors:** Andrew Spiel, Amber Shada, Luis Lomeli

**Affiliations:** 1 Division of Gastroenterology and Hepatology, Department of Medicine, University of Wisconsin School of Medicine and Public Health, Madison, WI; 2 Department of Surgery, University of Wisconsin School of Medicine and Public Health, Madison, WI

## CASE REPORT

A 58-year-old man presented with dizziness, weakness, and chronic diarrhea. He had a history of rheumatoid arthritis and a perforated peptic ulcer for which he underwent a Billroth II gastrectomy in 1983. He was a former smoker and used nonsteroidal anti-inflammatory drugs. For the past 8 months, he had been having up to 15 episodes of diarrhea per day with undigested food particles, significantly worse postprandially. He had lost approximately 30 lbs over this time frame.

The patient had been studied for infectious causes of diarrhea and had undergone computed tomography (CT) imaging 6 weeks after admission with oral contrast that was unrevealing. An esophagogastroduodenoscopy and colonoscopy 4 months after his symptoms had showed a marginal ulceration at the gastrojejunal anastomosis with biopsies negative for dysplasia. Additional random gastric, small bowel and colonic biopsies were negative for *Helicobacter pylori*, villous atrophy, and microscopic colitis. A hydrogen breath test was positive 2 weeks before admission, and he was treated for small intestinal bacterial overgrowth with a 10-day course of ciprofloxacin.

**Figure 1.**
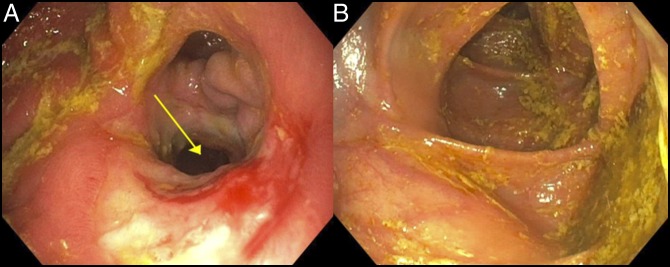
Esophagogastroduodenoscopy and colonoscopy showing a large gastrocolic fistula with endoscopic passage easily into the transverse colon.

**Figure 2.**
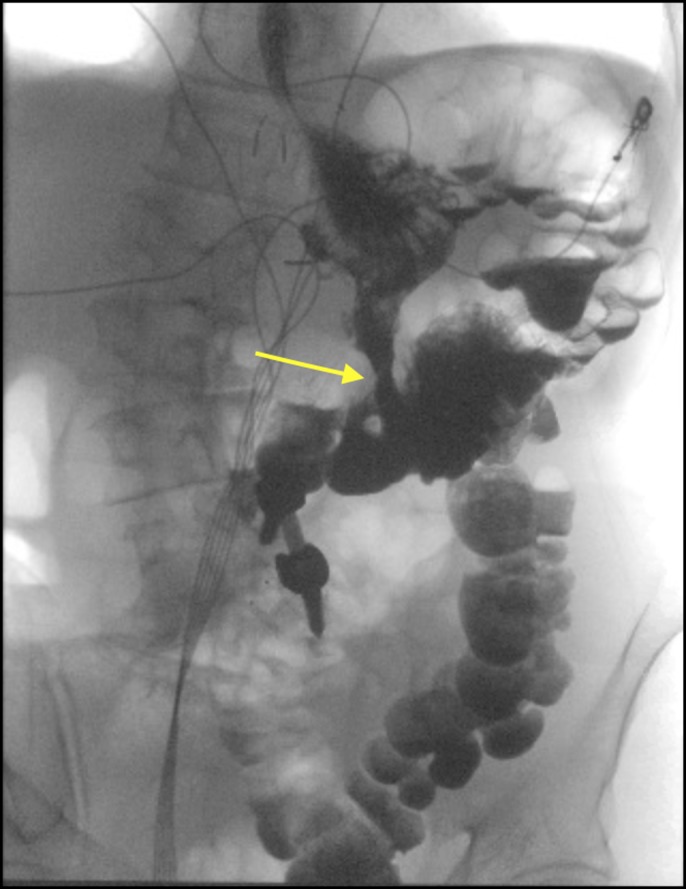
An upper gastrointestinal series showing a gastrocolic fistula with preferential filling of the gastric contents into the transverse colon.

On admission, laboratory analysis was significant for hypokalemia to 2.4 mmol/L and hypoalbuminemia to 1.9 g/dL. The patient underwent repeat esophagogastroduodenoscopy and colonoscopy showing a large gastrocolic fistula with endoscopic passage into the transverse colon (Figure 1). Confirmation of gastrocolic fistula was made with an upper gastrointestinal series showing a gastrocolic fistula with preferential filling of the gastric contents into the transverse colon (Figure 2).

Gastrocolic fistula is a rare late complication of Billroth II gastrectomy for peptic ulcer disease. Given the infrequent nature of this complication, the diagnosis can often be delayed.^1^ The diagnosis is typically made on endoscopy and contrast-enhanced CT or magnetic resonance imaging. Our patient's initial contrast-enhanced CT did not reveal the etiology. This could be in part due to the timing of contrast. The pathogenesis of the fistula is typically considered a late representation of a chronic stromal ulcer.^2^ In this case, chronic nonsteroidal anti-inflammatory drug use for rheumatoid arthritis symptoms is suspected as the inciting etiology for ulcer development. The typical patient presentation includes malnutrition, electrolyte derangements, weight loss, and diarrhea.^3^ Obtaining biopsies of the adjacent gastric mucosa and colonoscopy can rule out malignancy and inflammatory bowel disease as potential etiologies, as it did in this case. He underwent endoscopic suturing with the repair of the gastrocolic defect 3 months after nutritional optimization with J-tube feedings. Unfortunately, follow-up upper gastrointestinal series showed a persistent fistula. He is scheduled for laparoscopic repair.

## DISCLOSURES

Author contributions: A. Spiel wrote the manuscript and is the article guarantor. A. Shada reviewed the literature. L. Lomelli reviewed the literature and edited the manuscript.

Financial disclosure: None to report.

Informed consent was obtained for this case report.
